# Supplementary material for: Homology modeling and docking studies of a Δ9-fatty acid desaturase from a Cold-tolerant Pseudomonas sp. AMS8
Source: PeerJ. 2018 Mar 19;6:e4347. doi: 10.7717/peerj.4347 (PMC5863719; doi:10.7717/peerj.4347)
Supplement: Supplemental Information 1 [file peerj-06-4347-s002.docx]

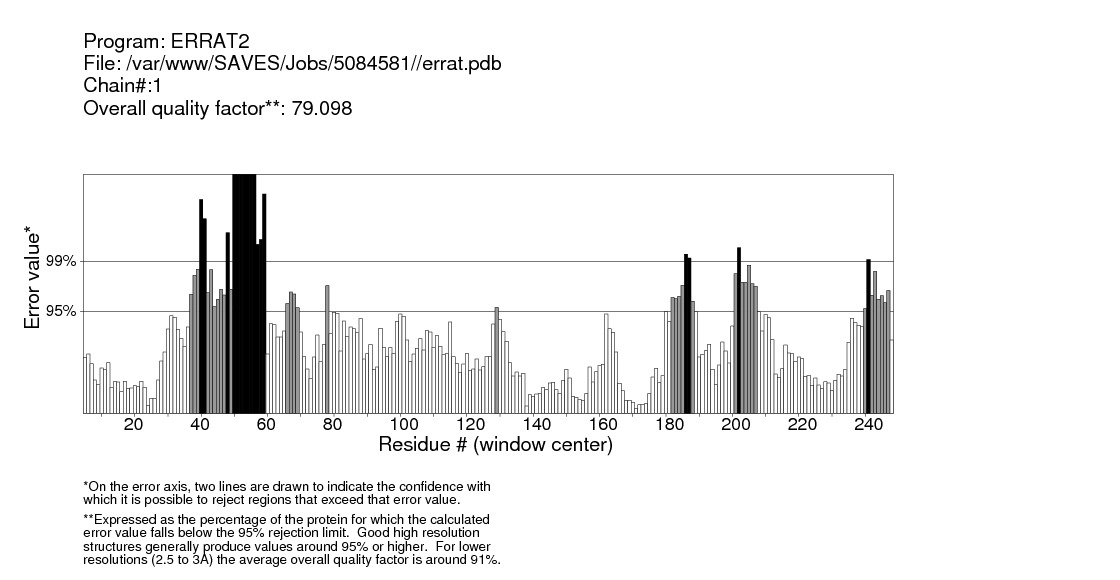


Raw data: Quality verification plot of the energy minimized model (AMX8-em1) of the ∆9-fatty acid desaturase performed using ERRAT. The two lines drawn on the error axis show the confidence with which it is possible to reject regions that exceed that error value. Good high resolution structures generally produce values around 95% or higher whereas lower resolution (2.5- -3Å) have an average overall quality factor around 91%.


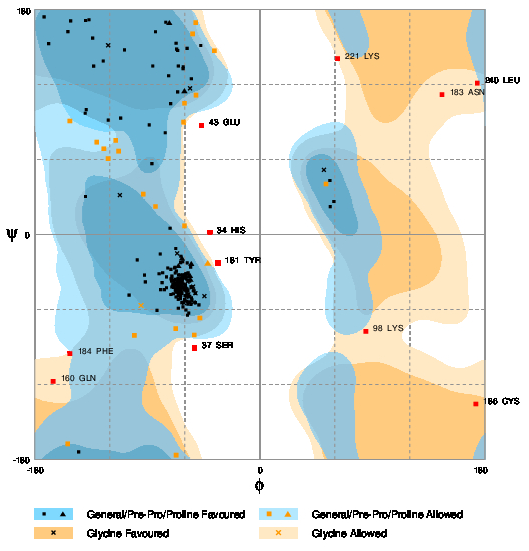


## Evaluation of residues

Residue [ 7 :LEU] ( -83.39, 22.36) in Allowed region

Residue [ 8 :ASP] (-130.40, 73.84) in Allowed region

Residue [ 10 :SER] ( -60.98, 89.94) in Allowed region

Residue [ 33 :LEU] ( -60.24, 6.87) in Allowed region

Residue [ 35 :ARG] (-100.35, -80.71) in Allowed region

Residue [ 36 :TYR] ( -67.23, -75.26) in Allowed region

Residue [ 63 :ALA] ( 52.96, 40.36) in Allowed region

Residue [ 65 :ARG] ( -53.75, 160.46) in Allowed region

Residue [ 66 :GLU] ( -51.21, 169.54) in Allowed region

Residue [ 80 :THR] (-124.74, 68.56) in Allowed region

Residue [ 82 :ASP] (-121.27, 60.66) in Allowed region

Residue [ 88 :VAL] (-112.91, 66.69) in Allowed region

Residue [ 92 :LEU] ( -66.86,-176.49) in Allowed region

Residue [ 99 :GLY] ( -95.01, -56.74) in Allowed region

Residue [ 103 :TYR] (-115.19, 75.28) in Allowed region

Residue [ 118 :LYS] ( -47.91, -66.78) in Allowed region

Residue [ 134 :PRO] ( -41.61, -22.73) in Allowed region

Residue [ 185 :GLU] (-151.87, 90.80) in Allowed region

Residue [ 199 :ILE] ( -93.22, 32.25) in Allowed region

Residue [ 200 :VAL] ( -52.42, -80.38) in Allowed region

Residue [ 220 :VAL] ( -51.03, 111.52) in Allowed region

Residue [ 224 :GLU] ( -60.17, 104.96) in Allowed region

Residue [ 239 :ARG] (-153.64,-167.47) in Allowed region

Residue [ 247 :ALA] ( -36.30, 146.98) in Allowed region

Residue [ 34 :HIS] ( -39.91, 1.52) in Outlier region

Residue [ 37 :SER] ( -52.05, -90.69) in Outlier region

Residue [ 43 :GLU] ( -46.42, 87.37) in Outlier region

Residue [ 98 :LYS] ( 84.92, -77.47) in Outlier region

Residue [ 160 :GLN] (-165.27,-117.55) in Outlier region

Residue [ 181 :TYR] ( -33.51, -22.81) in Outlier region

Residue [ 183 :ASN] ( 145.83, 111.91) in Outlier region

Residue [ 184 :PHE] (-151.84, -95.34) in Outlier region

Residue [ 186 :CYS] ( 172.89,-135.57) in Outlier region

Residue [ 221 :LYS] ( 62.40, 140.72) in Outlier region

Residue [ 240 :LEU] ( 174.01, 121.09) in Outlier region

Number of residues in favoured region (~98.0% expected) : 214 ( 85.9%)

Number of residues in allowed region ( ~2.0% expected) : 24 ( 9.6%)

Number of residues in outlier region : 11 ( 4.4%)

Raw data: Ramachandran plot of *Pseudomonas* sp. AMS8 ∆9-fatty acid desaturase model (AMX8-em1) generated by RAMPAGE server.


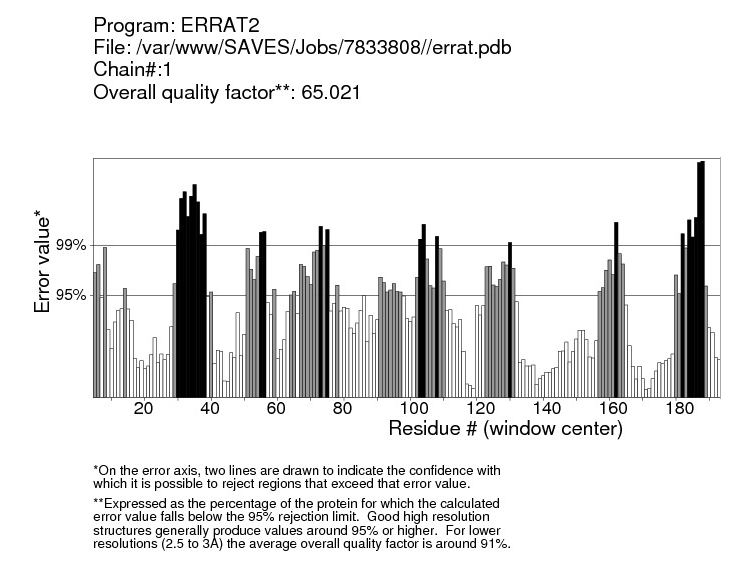


Raw data: Quality verification plot of the energy minimized model (AMX8-em2) of the ∆9-fatty acid desaturase performed using ERRAT. The two lines drawn on the error axis show the confidence with which it is possible to reject regions that exceed that error value. Good high resolution structures generally produce values around 95% or higher whereas lower resolution (2.5- -3Å) have an average overall quality factor around 91%.


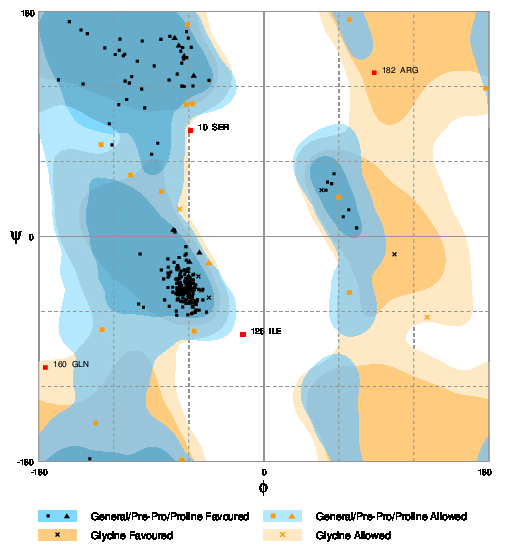


**Evaluation of residues**

Residue [ 47 :GLY] ( 130.71, -64.45) in Allowed region

Residue [ 63 :ALA] ( 59.76, 31.53) in Allowed region

Residue [ 65 :ARG] ( -65.54,-178.63) in Allowed region

Residue [ 77 :LYS] (-130.07, 73.54) in Allowed region

Residue [ 85 :THR] (-106.51, 49.19) in Allowed region

Residue [ 86 :VAL] ( -57.04, 106.09) in Allowed region

Residue [ 90 :LYS] ( 68.44, 173.56) in Allowed region

Residue [ 95 :TYR] ( 68.46, -44.82) in Allowed region

Residue [ 103 :TYR] (-134.41,-149.25) in Allowed region

Residue [ 134 :PRO] ( -43.91, -20.77) in Allowed region

Residue [ 162 :MET] ( -55.58, -75.71) in Allowed region

Residue [ 180 :GLY] ( -67.37, 21.94) in Allowed region

Residue [ 183 :ASN] ( 177.50, 118.62) in Allowed region

Residue [ 184 :PHE] (-129.31, -74.52) in Allowed region

Residue [ 190 :ALA] ( -61.10, 169.36) in Allowed region

Residue [ 205 :LEU] ( -81.92, 35.97) in Allowed region

Residue [ 224 :GLU] ( -62.01, 105.63) in Allowed region

Residue [ 10 :SER] ( -58.52, 84.81) in Outlier region

Residue [ 125 :ILE] ( -16.49, -78.37) in Outlier region

Residue [ 160 :GLN] (-174.67,-104.78) in Outlier region

Residue [ 182 :ARG] ( 88.42, 131.00) in Outlier region

Number of residues in favoured region (~98.0% expected) : 228 ( 91.6%)

Number of residues in allowed region ( ~2.0% expected) : 17 ( 6.8%)

Number of residues in outlier region : 4 ( 1.6%)

Raw data: Ramachandran plot of *Pseudomonas* sp. AMS8 ∆9-fatty acid desaturase model (AMX8-em2) generated by RAMPAGE server.
